# Does self-administered vaginal misoprostol result in cervical ripening in postmenopausal women after 14 days of pre-treatment with estradiol? Trial protocol for a randomised, placebo-controlled sequential trial*

**DOI:** 10.1111/j.1471-0528.2008.01727.x

**Published:** 2008-06

**Authors:** KS Oppegaard, M Lieng, A Berg, O Istre, E Qvigstad, B-I Nesheim

**Affiliations:** a Department of Gynaecology, Helse Finnmark, Klinikk HammerfestHammerfest, Norway; b Department of Gynaecology, Women and Children's Division, Ullevål University HospitalKirkeveien, Oslo, Norway; c Faculty of Medicine, University of Oslo and Department of Gynaecology, Women and Children's Division, Ullevål University HospitalKirkeveien, Oslo, Norway; d Faculty of Medicine, University of Oslo and Department of Obstetrics, Women and Children's Division, Ullevål University HospitalKirkeveien, Oslo, Norway

**Keywords:** Cervical ripening, estradiol, hysteroscopy, misoprostol, postmenopausal

## Abstract

**Objective:**

To compare the impact of 1000 micrograms of self-administered vaginal misoprostol versus self-administered vaginal placebo on preoperative cervical ripening after pre-treatment with estradiol vaginal tablets at home in postmenopausal women prior to day-care operative hysteroscopy.

**Design:**

Randomised double-blind placebo-controlled sequential trial. The boundaries for the sequential trial were calculated on the primary outcomes of a difference of cervical dilatation ≥1 millimetre, with the assumption of a type 1 error of 0.05 and a power of 0.95.

**Setting:**

Norwegian university teaching hospital.

**Population:**

Postmenopausal women referred for day-care operative hysteroscopy.

**Methods:**

The women were randomised to either 1000 micrograms of self-administered vaginal misoprostol or self-administered vaginal placebo the evening before day-care operative hysteroscopy. All women had administered a 25-microgram vaginal estradiol tablet daily for 14 days prior to the operation.

**Main outcome measures:**

Preoperative cervical dilatation (difference between misoprostol and placebo group, primary outcome), difference in dilatation before and after administration of misoprostol or placebo, number of women who achieve a preoperative cervical dilatation ≥5 millimetres, acceptability, complications and side effects (secondary outcomes).

**Results:**

Intra-operative findings and distribution of cervical dilatation in the two treatment groups: values are given as median (range) or *n*(%). Difference in dilatation before and after administration of misoprostol and placebo: values are given as median (range) of intraindividual differences. Percentage of women who achieve a cervical dilatation of ≥5 mm, percentage of women who were difficult to dilate. Acceptability in the two treatment groups: values are given as completely acceptable *n*(%), fairly acceptable *n*(%), fairly unacceptable *n*(%), completely unacceptable *n*(%). Pain in the two treatment groups: pain was measured with a visual analogue scale ranging from 0 (no pain) to 10 (unbearable pain): values are given as median (range). Occurrence of side effects in the two treatment groups. Values are given as *n*(%). Complications given as *n*(%).

**Funding sources:**

No pharmaceutical company was involved in this study. A research grant from the regional research board of Northern Norway has been awarded to finance Dr K.S.O.'s leave from Hammerfest hospital as well as travel expenses between Hammerfest and Oslo, and research courses. The research grant from Prof B.I.N. (Helse Øst) funded the purchase of estradiol tablets, the manufacturing costs of misoprostol and placebo capsules from the hospital pharmacy, as well as the costs incurred for preparing the randomisation schedule and distribution of containers containing capsules to hospital. Prof B.I.N.'s research grant also funded insurance for the study participants.

**Conclusions:**

Estimated completion date 31 December 2008.

## Background

Over the past 20 years, minimally invasive operative techniques have been introduced for treating intrauterine pathology. Operative hysteroscopy or resectoscopy is currently the most common method in the industrialised world for treating intrauterine pathology, such as leiomyomas and endometrial polyps. Endometrial resection is a standard treatment for abnormal uterine bleeding, if less invasive procedures fail.^1^ The diameter of resectoscopes (usually Charriere 24 or 26) necessitate dilatation of the cervical canal to 10 or 11 millimetres prior to insertion of the instrument. The possible complications encountered during dilatation, such as cervical tears, creation of false passages and uterine perforation are reported to be mainly related to difficulty of cervical dilatation in nulliparous and postmenopausal women.^2^ An audit of women who have undergone operative hysteroscopy in our department supports this,^3^ showing a complication rate of 7.8% related to hysteroscopic resection of endometrial polyps. In our recent study^4^ the frequency of complications during operative hysteroscopy was 11% and occurred mostly in nulliparous premenopausal or postmenopausal women. Experienced gynaecologists judged 42% of dilatations in postmenopausal women to be difficult.

Reduction in incidence of cervical injury and uterine perforation during termination of pregnancy has been demonstrated by preoperative cervical ripening^5^,^6^ and may be achieved either mechanically, with osmotic dilators,^7^ or biochemically with prostaglandins.^8^ Locally applied prostaglandins are an inexpensive and resource-saving method of achieving cervical ripening compared with osmotic dilators, particularly if women are able to insert the tablets vaginally themselves at home prior to their operation. Misoprostol is a synthetic prostaglandin E_2_ analogue that is widely used for this purpose as it is inexpensive and effective.^8^,^9^ Misoprostol appears to be effective for cervical ripening in nonpregnant premenopausal women prior to hysteroscopy.^10^ Further research in postmenopausal women has been recommended, to determine whether misoprostol is effective in cervical ripening in this population. We found six reported randomised controlled trials published in English (Appendix 1,2) that evaluated the efficacy of misoprostol on cervical ripening on postmenopausal women published before 31 October 2007, after searching medical literature databases including Pubmed^11^ and EMBASE Ovid.^12^ The search terms used included ‘misoprostol’, ‘cervical ripening/priming’‘hysteroscopy’, and ‘operative hysteroscopy’. References from identified publications were manually searched and cross-referenced to identify additional relevant articles. The studies have shown different cervical response and outcomes.^13^–^18^ In our previous study^4^ we found that 1000 micrograms of self-administered vaginal misoprostol at home at least 12 hours prior to operative hysteroscopy was effective, safe and acceptable for cervical priming in premenopausal but not in postmenopausal women. We speculate whether a lack of estrogen in postmenopausal women is the main reason why misoprostol does not have a significant cervical priming effect.

The mediators of the cervical ripening process are still largely unknown. They have been studied in premenopausal women^19^–^23^ and it has been demonstrated that local application of prostaglandin E_2_ induces cervical ripening by increased remodelling of the cervical connective tissue.^21^ Estrogen also enhances cervical ripening.^23^ Locally applied estrogen is effective for vaginal atrophy in postmenopausal women.^24^ It is probably necessary to treat postmenopausal women who have not previously used hormone therapy with daily locally applied estrogen for 2 weeks to achieve a sufficient effect on the vaginal and urethral epithelium.^25^ There are no published studies available on the effect of locally applied estrogen on cervical tissue and epithelium. We have found only one previous study that has investigated the ripening effect of misoprostol on the estrogen-pretreated cervix in postmenopausal women.^18^ The authors concluded that misoprostol alone was not effective for cervical ripening, but that it was effective after 22 women had first used estriol vaginal cream for 14 days. However, the study was neither designed nor conducted in accordance with the recommendations in the CONSORT statement^26^ and lacked a sample size calculation with regards to evaluating primary outcome measures. Furthermore, misoprostol was not compared to a placebo and estriol cream was compared to clindamycine phosphate cream.

The aim of our study is to investigate whether 1000 micrograms of self-administered vaginal misoprostol 12 hours before operative hysteroscopy results in effective preoperative cervical ripening after two weeks pre-treatment with 25 micrograms daily vaginal estradiol, compared with placebo (lactosum monohydricum) in postmenopausal women.

## Hypothesis

Null hypothesis: there is no clinically significant difference in preoperative baseline cervical dilatation (<1 mm), between postmenopausal women who receive vaginal misoprostol and postmenopausal women who receive vaginal placebo, after pre-treatment with estradiol.

## Interventions

All consecutive postmenopausal women (defined at 1 year after last menstruation) referred for day-care operative hysteroscopy, and who have given informed consent, will be eligible for study recruitment. All women included in the study, but not using hormone therapy with estrogen, will start daily treatment with estradiol vaginal tablets (Vagifem®, Novo Nordisk, Måløv, Denmark, containing 25 micrograms of estradiol per tablet) 2 weeks prior to the operation. The effect of self-inserted 1000-microgram vaginal misoprostol 12 hours before operative hysteroscopy on cervical ripening will be compared to self-inserted vaginal placebo 12 hours before operative hysteroscopy.

## Randomisation procedure

The women will be assigned to either 1000-microgram vaginal misoprostol or vaginal placebo. Full randomisation at a ratio of 1:1 was created on 23 November 2007 with the randomisation plan generator, as described by G. Dallal.^27^ The randomisation procedure is a third party concealed randomisation, performed by the hospital pharmacist.

## Outcome measures

### Primary end-point

- The primary outcome is the preoperative baseline cervical dilatation in the two treatment groups.

### Secondary end-points

- Difference between baseline cervical dilatation at recruitment, before treatment with estradiol, misoprostol or placebo, and preoperative dilatation.
- The number of women who achieve satisfactory cervical priming (cervical dilatation ≥5 mm). Five millimetres is chosen as ‘satisfactory’, as this would permit insertion of a diagnostic hysteroscope without further dilatation. A preoperative cervical dilatation of 5 millimetres would also make it much easier to further dilate the cervix with Hegar dilators if necessary, decreasing the risk of creating a false passage.
- Acceptability of self-administration of vaginal capsules at home.
- Number of dilatations judged as ‘easy’ or ‘difficult’.
- Frequency of complications.

## Number of women required

**Figure 1:**
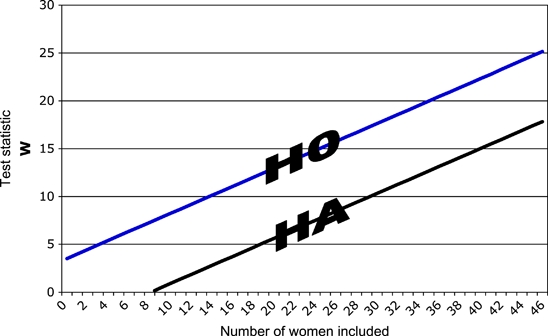
Continuation region of the sequential test. The stopping boundaries are shown. H0, boundary for the null hypothesis, HA, boundary for the alternative hypothesis.

For ethical reasons it is important to keep the number of women needed to reach a conclusion in clinical trials as low as possible. A sequential trial plan using a two-sample sequential Wilcoxon test developed by Skovlund and Walløe is therefore used.^28^–^30^ This method has previously been used in relatively few clinical trials, but has been shown to be easy to use and robust. It is expected to reduce the number of women needed to reach a conclusion, compared with the number required in a corresponding fixed sample trial.^31^,^32^ In our previous trial we needed only 20% of the sample size before reaching a conclusion on postmenopausal women using this method. The analysis was tailored to reach a conclusion on the primary outcome as soon as the difference was significant, so that as few women as possible were enrolled. A trial with a fixed sample size, with the same primary end-point as ours, would have required 105 postmenopausal women (SD 2.0) analysed in order to obtain a power of 95% based on a *t* test, assuming normally distributed observations and equal variance in the two groups. The boundaries needed for the statistical model were calculated on the preoperative variability (SD) in cervical dilatation from our previous trial (Figure 1). The mean cervical dilatation was 4.2 millimetres, SD 2.0. The design of the main trial is based on a significance level of 5% and a power of 95% against a 1-millimetre difference in the cervical dilatation caused by misoprostol and placebo. As it seems unlikely that use of misoprostol could cause a constriction of the cervix, a one-sided test is chosen. The trial will continue until either the null hypothesis or the alternative hypothesis has been refuted. Following the sequential Wilcoxon test by Skovlund and Walløe, the estimator of normal means is applied, if the observations are normally distributed.^33^

## Eligibility criteria

*Inclusion criteria:* All postmenopausal (>1 year since last menstruation) women who are referred to day-care hysteroscopy with a medical indication for hysteroscopy, and who have given informed consent, will be eligible for study recruitment. Exclusion criteria: women who do not wish to participate, women who are medically unfit for hysteroscopy, women who are medically unfit for participation in any clinical trial, women who do not have a medical indication for hysteroscopy, women who have previously had, or currently have breast or gynaecological cancer, women who have a medical contraindication for locally applied estradiol, women who are currently using hormone therapy, women who are unable to communicate in Norwegian and women with a known allergy to misoprostol.

## Trial process and data collection

**Figure 2:**
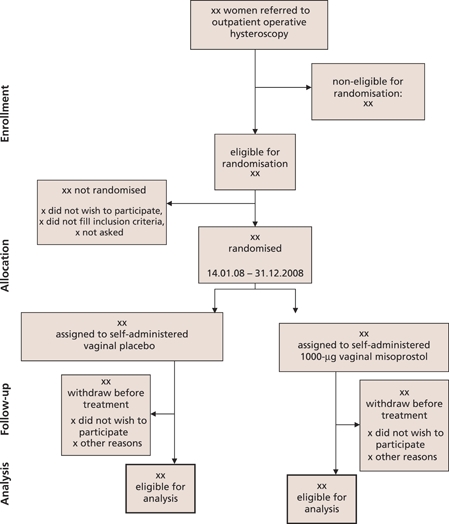
Flow diagram.

The present study protocol is designed according to the recommendations in the CONSORT statement^26^ and will be submitted to BJOG for review before recruitment of women. The study is a randomised, controlled, double-blind one-centre study at a Norwegian central university gynaecological outpatients department. The study flow (Figure 2) is planned as follows:

### Enrollment

All women referred to day-care operative hysteroscopy at Ullevål University Hospital in Oslo, Norway, will be sent an invitation to be included in the study. Women are referred to this hospital for operative hysteroscopy from private practising gynaecologists, GPs or other hospitals. The common presenting complaints in postmenopausal women are postmenopausal uterine bleeding and discovery of clinically silent endometrial polyps. The invitation for study participation will be sent together with dates for a preoperative outpatient consultation. The study invitation will include detailed information regarding the study, as well as an informed consent form. The outpatient consultation will take place shortly after the referral has been received. Here, the women will receive additional information and be given the option to participate in the study.

### Allocation

M.L. will be responsible for examining women at the outpatient consultation and for recruiting the women. Women who elect to participate in the study, and who are not already using estrogen hormone therapy, will receive estradiol vaginal tablets daily for 2 weeks use prior to operative hysteroscopy, as well as either 1000-microgram misoprostol or placebo according to the randomisation list, to be self-inserted vaginally at least 12 hours before operative hysteroscopy. Before an endometrial biopsy and cervical smear is taken as part of the routine preoperative examination, cervical dilatation will be measured by passing Hegar dilators through the cervix in ascending order starting with Hegar dilator of size 2 millimetres. No other tests will be taken for evaluation as part of the trial, but serum haemoglobin, or other clinical procedures, such as an electrocardiogram, may be ordered as part of the routine preoperative examinations prior to hysteroscopy.

The women will receive an appointment for their operation at least 4 weeks after the initial outpatient consultation; the length of the waiting period will depend on the suspected diagnosis and the women's symptoms and signs. Any woman with a malignant histology result, or with clinical symptoms and signs that strongly indicate a malignant disease will either be excluded from study participation upon examination, or excluded as soon as the histology report is presented (usually within a week). They will thus be prevented from using either misoprostol or estrogen. Placebo misoprostol tablets are difficult to make, therefore the hospital pharmacist will manufacture gelatine capsules with an identical appearance. The active misoprostol will be ground up as a whitish powder inserted into gelatine capsules (500-microgram misoprostol per capsule), as well as an inactive ingredient, lactosum monohydricum—which has an identical appearance to ground misoprostol tablets—and subsequently inserted into gelatine capsules as placebo. The hospital pharmacist will thereafter prepare numbered, opaque, sealed plastic containers labelled ‘Misoprostol 0.5 mg/Placebo, 2 vaginal capsules’. The prepared capsules will be inserted into containers by the hospital pharmacist, who will then seal them with tamper-proof seals. Each container will contain two capsules. Half of the containers will contain two capsules with 500-microgram misoprostol each, while the other half will contain two placebo capsules. The containers will then be delivered to the outpatients clinic. As the women are recruited at the outpatient consultation, M.L. will record the preoperative variables on a standardised case report form (on page 1) and the women will be given a plastic container containing the capsules. In addition, each woman will be given a prescription for 15 tablets of 25-microgram estradiol to be collected at the hospital pharmacy. Hence, those involved in administering the intervention and the women will be blinded to the treatment received. Each study participant will open a numbered container at home, containing either misoprostol, or lactosum monohydricum in capsules. The women will be instructed to begin inserting one estradiol vaginal tablet daily 14 days before their operation. In addition, they will be instructed to insert the misoprostol/placebo capsules vaginally, as deep as possible, after voiding urine at approximately 9 p.m. the evening before the operation.

### Follow up

On admission to the operating theatre, nurses will record symptoms and comments from the women on the case report form (page 1). The women will record pain experienced between the insertion of capsules and the operation on a visual analogue scale score, ranging from 0 (no pain) to 10 (unbearable pain).

Women will be asked to rate acceptability of self-administered capsules on a scale from 1 to 4 (1, completely acceptable; 2, fairly acceptable; 3, fairly unacceptable; 4, completely unacceptable). The women will then be given a general intravenous anaesthetic (propofol/fentanyl/alfentanyl) by the attending nurse anaesthetist, after which the theatre nurses will prepare the women for operation by disinfecting the vulval and vaginal area with a 0.05% chlorhexidine acetate solution (Fresenius Kabi, Halden, Norway). Visible vaginal capsule remains will be noted before the area is irrigated. The case report form with the preoperative variables, recorded symptoms and comments from the women on page 1 will then be turned over to page 2 so that this information is not available to either M.L. or the operating gynaecologist. The operators will then be summoned to the operating theatre. The women's faces are hidden from the operators. The operators should thus both be blinded to which treatment the women had received and to the occurrence of possible side effects from the treatment, as recorded by the nurses.

### Analysis

We will try to ensure that only a single gynaecologist performs all the operative hysteroscopies. M.L. will instruct the operator as to how to correctly assess preoperative cervical dilatation, so that they are in agreement with the measurement. We hope that this might reduce subjectivity in gauging preoperative cervical dilatation. During our previous trials, the use of several gynaecologists assessing cervical dilatation may have increased the risk of inter-observer variability. The use of a single gynaecologist in this trial would reduce this interdoctor assessment variation. Before the operative hysteroscopy, the operator will measure the preoperative degree of cervical dilatation by passing Hegar dilators through the cervix in ascending order starting with a size of 2 millimetres. The size of the largest dilator passed into the inner cervical ostium without subjective resistance felt by the operator will be recorded as the preoperative degree of dilatation. If there is initial resistance with Hegar dilator of size 2 millimetres, the result will be recorded as 0 millimetres. After the cervical canal is dilated to a Hegar dilator of size 10 or 11 millimetres, an Olympus (Olympus, Hamburg, Germany) rigid resectoscope model A22026A (Charriere 26) equipped with a Hopkins 12° rigid fibreoptic model A22001A, will be passed into the uterine cavity. A sodium chloride 9% solution (Baxter, Norfolk, UK) will be infused for uterine irrigation. A bipolar diathermal current of 280 watts (pure cut) supplied by a Surgmaster® US-40 (Olympus) diathermia unit will routinely be used for resection of pathological uterine masses (myomas, polyps, uterine septae etc.) and endometrium. For haemostasis coagulation, a current of 80 watts will be applied. Adverse events during the operation, such as superficial cervical lacerations, production of false passage of the cervix during cervical dilatation and perforation of the uterus will be recorded.

The case report form will then be delivered to K.S.O and B.I.N. for separate and parallel analysis of the primary outcome using the sequential method. In this case, crossing the lower boundary will mean that misoprostol is significantly superior to placebo and crossing the upper boundary will mean that the two treatments are equally effective. After a conclusion has been reached on the primary outcome, K.S.O. and M.L. will analyse the data for secondary outcomes, but without performing significance tests, because the trial is neither designed nor powered for these.

### Discontinuation

After recruitment, women may withdraw from the trial if they do not wish to participate. They may decline to take the prescribed estradiol vaginal tablets, misoprostol/placebo capsules, or have the hysteroscopy itself. Side effects from vaginal estradiol tablets are generally mild and well tolerated, but the women may stop taking them if they feel that these are unacceptable. (The misoprostol/placebo regimen is a once-only dosage.) Acute illness would necessitate postponement of the operation and would mean that the women would have to be excluded from analysis.

### Registering and reporting of suspected adverse reactions

Any unexpected and serious preoperative side effects and suspect adverse reactions will be immediately reported to K.S.O. or B.I.N., as well as the trial steering committee (see the section ‘Trial steering committee’). The Norwegian Medicines Agency (SLV) and regional ethics committee will also be notified. Procedure-related complications and side effects will be continuously monitored and reported. The women will be contacted by telephone–and the patient records will be reviewed—after 14 days for registration of postoperative complications.

Start date was 14 January 2008.

Expected completion date is 31 December 2008.

## Data collection (variables)

Pre- intra-, and postoperative findings in the two treatment groups.

**Table:** Preoperative findings (recorded at preoperative consultation)

	Vaginal placebo (*n* = xx)	1000-microgram vaginal misoprostol (*n* = xx)
Age, height and weight		
Parity		
Years of menopause		
No. births/previous vaginal delivery		
Previous cone biopsy		
Baseline cervical dilatation before treatment		
Indications for operative hysteroscopy

**Table:** Preoperative findings (recorded by nurse on the day of the operative hysteroscopy prior to the operation)

	Vaginal placebo (*n* = xx)	1000-microgram vaginal misoprostol (*n* = xx)
Duration of vaginal capsules given before operation (hours)		
Preoperative side effects (e.g. nausea, diarrhoea, vaginal bleeding, cramps)		
Pain measured on a visual analogue scale score 0–10		
Capsule treatment acceptability (1 = completely acceptable; 2 = fairly acceptable; 3 = fairly unacceptable; 4 = completely unacceptable)		
Any comments from patient

**Table:** Intraoperative findings (recorded by operating gynaecologist)

	Vaginal placebo (*n* = xx)	1000-microgram vaginal misoprostol (*n* = xx)	*P* value
Markedly anteverted or retroverted uterus			
Median baseline cervical dilatation (mm)			
No. women achieving preoperative cervical dilatation ≥5 mm			
Dilatation time (minutes)			
Dilatation difficult (yes/no)			
Complications (e.g. cervical laceration, false passage of the cervix, uterine perforation)			
Operative procedure			
Difference in dilatation before and after administration of estradiol and misoprostol or placebo

**Table:** Post-operative findings (recorded by one of the study authors)

	Vaginal placebo (*n* = xx)	1000-microgram vaginal misoprostol (*n* = xx)	*P* value
Recorded postoperative complications			

## Statistical analysis plan

### Primary outcome

Sequential method by Skovlund and Walløe. See section ‘Number of patients required’.

### Secondary outcomes

SPSS will be used to analyse secondary outcomes.

## Publication policy

The study protocol will be sent to BJOG for peer review prior to inclusion of women. The manuscript will be sent to BJOG upon completion of the data analysis.

## Trial steering committee

Bjørn Busund (Head of Department, Women and Children's Division, Ullevål University Hospital), Nina Kristoffersen (Clinical Study Coordinator, Ullevål Hospital Pharmacy), Eva Skovlund (Senior adviser, Norwegian Medicines Agency. Professor II, University of Oslo).

## Data monitoring and ethics committee

Ethical approval from The Regional Committee for Medical Research Ethics (Regional komité for medisinsk forskningsetikk, Nord-Norge, REK Nord) in Northern Norway^34^ was obtained on 16 October 2007 (Ref no. 200704112-5/MRO/400). Permission from the Norwegian Medicines Agency^35^ (Statens Legemiddelverk, SLV) was granted on 6 December 2007 (Ref no. 07/12515-8) and from Ullevål Hospital's Advisory Committee on the Protection of Patient Records (Personvernansvarlig) on 17 December 2007. The study protocol is published on http://www.clinicaltrials.gov, and has been submitted to the European Clinical Trials Database and BJOG. Each participating woman in the study will sign an informed consent, and will be insured through the Drug Liability Association (Legemiddelforsikringen) with liability insurance in connection with clinical trials of drugs.

## Centres

The Women's Clinic at Ullevål University Hospital, Oslo, Norway.

## Funding source

No pharmaceutical company was involved in this study. The funding sources will have no input into the study design, collection, analysis or interpretation of the data, report preparation or in the decision to submit the paper for publication. K.S.O, M.L. and B.I.N. will have full access to all the data after women no longer are being recruited into the study and will have final responsibility to submit manuscripts for publication.

A research grant from the regional research board of Northern Norway has been awarded to finance Dr K.S.O.'s leave from Hammerfest hospital as well as travel expenses between Hammerfest and Oslo, and research courses. The research grant from Prof B.I.N. (Helse Øst) will fund the purchase of estradiol tablets, the manufacturing costs of misoprostol and placebo capsules from the hospital pharmacy, as well as the costs incurred for preparing the randomisation schedule and distribution of containers containing capsules to hospital. Prof B.I.N.'s research grant will also fund insurance for the study participants.

Ullevål University Hospital's personnel will send invitations for study participation to the women and ensure that the data collection is processed according to the recommendations in the CONSORT statement.^26^

## Contribution to authorship

K.S.O. was chief investigator, guarantor, responsible for original protocol, data analysis, and grant holder responsible for financing extra costs in addition to the normal running costs of the department, responsible for applications to Regional Ethics Committee, The Norwegian Medicines Agency, European Clinical Trials Database and for publishing the protocol on clinicaltrials.gov, drafting, revising, responsible for final report and scientific papers. M.L. was responsible for ascertainment of women, treatment and assessments over the trial period, data analysis, revising and final approval. A.B. was responsible for operating on the women, assessment of the primary outcome in conjunction with M.L., revision and final approval. O.I. was responsible for logistical support, revision and final approval. E.Q. was responsible for original trial idea, logistical support, revision and final approval. B.I.N. was responsible for original protocol, trial methodology, data analysis, the application to Ullevål Hospital's Advisory Committee on the Protection of Patient Records, revision and final approval.

## Disclosure of interests

None are declared.

**Appendix 1:** Randomised controlled trials of misoprostol for cervical ripening on postmenopausal women (1)

Study ID	Reference	Sample size		Age (years)		Intervention	
				
		1	2	1	2	1	2
1	Ngai *et al.*(2001)^13^	18	16	56.1 (±4.9)	66.9 (±12.7)	oral misoprostol 400-μg 12 h before surgery	oral placebo (vitamin B6) 12 h before surgery
2	Fung *et al.*(2002)^14^	39	39	57.8 (±7.9), range 47–74	58.5 (±7.9), range 45–77	vaginal misoprostol 800-μg >5 h before surgery	vaginal placebo (thiamin) >5 h before surgery
3	Thomas *et al.*(2002)^15^	98	98	45.7 (±9.3)	48.1 (±9.3)	oral misoprostol 400-μg 12 or 24 h before surgery	oral placebo (not specified) 12 or 24 h before surgery
12	Bunnasathiansri *et al.*(2004)^16^	22	22	56.95 (±1.79)	55.68 (±1.22)	vaginal misoprostol 400-μg 6 h before surgery	vaginal placebo (vitamin B6) 6 h before surgery
14	Barcaite *et al.*(2005)^17^	51	54	56.1 (±8.8)	58.5 (±10.5)	vaginal misoprostol 400-μg 12 h before surgery	no agent
15	Atmaca *et al.*(2005)^18^	22	23	48.7 (±2.8)	48.6 (±1.9)	oral misoprostol 400-μg, 24 and 12 h before surgery, after 14 days vaginal estrogen, cream twice daily	oral misoprostol 400-μg, 24 and 12 h before surgery, after 14 days vaginal clindamycin, cream twice daily

**Appendix 2:** Randomised controlled trials of misoprostol for cervical ripening on postmenopausal women (2)

Study ID	Reference	Primary outcome		Dilatation time		Complications	
				
		1	2	1	2	1	2
1	Ngai *et al.*(2001)^13^	4.2 (±1.7) mm, 27.7 (±23.3) N	4.4 (±1.6) mm, 21.8 (±11.8) N	(operative time) 11.7 (± 10.8) min, range 4–41 min	6.4 (±4.2) min, 2–15 min	0%	0%
**Conclusion: no efficacy of oral misoprostol for cervical ripening (on postmenopausal women)**							
2	Fung *et al.*(2002)^14^	5.2 (±1.2) mm, range 3–7 mm	5.0 (±1.4) mm, range 3–9 mm	(operative time) 9.6 (±5.2) min, range 2–25 min	10.7 (±4.8) min, range 4–25 min	0%	0%
**Conclusion: no efficacy of vaginal misoprostol for cervical ripening (on postmenopausal women)**							
3	Thomas *et al.*(2002)^15^	6.9 (±2.2) mm	5.7 (±2.7) mm	1.14 (±1.3) min	1.39 (±1.1) min	19%	26.5%
						(cervical laceration) 4.1%	4.1%
						(perforation) 2%	2%
**Conclusion: oral misoprostol effective for cervical ripening (on premenopausal and postmenopausal women)**							
4	Bunnasathiansri *et al.*(2004)^16^	4.59 (±2.04) mm	4.41 (±1.62) mm	85 (±15–197) sec	60 (±10–300) sec	(uterine perforation) 9%	0%
**Conclusion: no efficacy of vaginal misoprostol for cervical ripening (on postmenopausal women)**							
5	Barcaite *et al.*(2005)^17^	7.6 (±1.4) mm	5.0 (±1.1) mm	(operative time) 22.2 (±6.8) min	22.3 (±7.6) min	0%	0%
**Conclusion: vaginal misoprostol effective for cervical ripening (on perimenopausal and postmenopausal women)**							
6	Atmaca *et al.*(2005)^18^	4.4 (±0.7) mm	3.7 (±0.7) mm	44.4 (±16.2) sec	61.4 (±18.3) sec	(uterine perforation) 0%	8.7%
**Conclusion: oral misoprostol effective for cervical ripening after pre-treatment with oestrogen cream (on postmenopausal women)**							

Min, minutes; N, Newtons; sec, seconds.

**Dummy Table 1:** Demographic characteristics of women in the two study groups according to dosage. Values are given as mean (SD) or *n*(%)

Characteristic	Self-administered vaginal placebo (*n* = xx)	Self-administered vaginal misoprostol (*n* = xx)
Age (y)		
BMI (kg/m^2^)		
Years of menopause		
Parity		
Previous vaginal delivery		
Previous cone biopsy

**Dummy Table 2:** Indications for operative hysteroscopy and operative procedure in the two study groups according to dosage

Indication	Self-administered vaginal placebo (*n* = xx)	Self-administered vaginal misoprostol (*n* = xx)
Procedure

**Dummy Table 3:** Intraoperative findings and distribution of cervical dilatation in the two treatment groups. Values are given as mean (SD), median (range) or *n*(%).

	Dosage group		P
		
	Self-administered vaginal placebo (*n* = xx)	Self-administered vaginal misoprostol (*n* = xx)	
Cervical dilatation (mm) (*n* = xx)			
Cervical dilatation (mm) pre-menopausal (*n* = xx)			
Cervical dilatation (mm) post-menopausal (*n* = xx)			
No. women achieving cervical dilatation ≥5 mm			
‘Difficult dilatation’			
Dilatation time (seconds)

**Dummy Table 4:** Preoperative side effects in the two treatment groups and findings during treatment

	Self-administered vaginal placebo (*n* = xx)	Self-administered vaginal misoprostol (*n* = xx)
Mean level of reported preoperative pain* (SD)		
Analgesic use *n*(%)		
Complications *n*(%)		
Occurrence of bleeding *n*(%)		
Other possible side effects		

* Measured with a VAS score, scale 0 (no pain)—100 unbearable pain.
